# Label-Free Multi Parameter Optical Interrogation of Endothelial Activation in Single Cells using a Lab on a Disc Platform

**DOI:** 10.1038/s41598-019-40612-8

**Published:** 2019-03-11

**Authors:** Damien King, MacDara Glynn, Sandra Cindric, David Kernan, Tríona O’Connell, Roya Hakimjavadi, Sinéad Kearney, Tobias Ackermann, Xavier Munoz Berbel, Andreu Llobera, Ulf Simonsen, Britt E. Laursen, Eileen M. Redmond, Paul A. Cahill, Jens Ducrée

**Affiliations:** 1 Dublin City University, School of Physical Sciences, National Centre for Sensor Research, Dublin, Ireland; 2 Dublin City University, School of Biotechnology, Irish Science Separation Cluster, Dublin, Ireland; 3 Dublin City University, School of Biotechnology, Vascular Biology & Therapeutics, Dublin, Ireland; 4 Centre Nacional de Microelectronica, Campus UAB, Barcelona, Spain; 5 Aarhus University, Department of Biomedicine, Aarhus, Denmark; 6 University of Rochester, Dept Surgery Rochester, New York, United States

## Abstract

Cellular activation and inflammation leading to endothelial dysfunction is associated with cardiovascular disease (CVD). We investigated whether a single cell label-free multi parameter optical interrogation system can detect endothelial cell and endothelial progenitor cell (EPC) activation *in vitro* and *ex vivo*, respectively. Cultured human endothelial cells were exposed to increasing concentrations of tumour necrosis factor alpha (TNF-α) or lipopolysaccharide (LPS) before endothelial activation was validated using fluorescence-activated cell sorting (FACS) analysis of inflammatory marker expression (PECAM-1, E-selectin and ICAM-1). A centrifugal microfluidic system and V-cup array was used to capture individual cells before optical measurement of light scattering, immunocytofluorescence, auto-fluorescence (AF) and cell morphology was determined. *In vitro*, TNF-α promoted specific changes to the refractive index and cell morphology of individual cells concomitant with enhanced photon activity of fluorescently labelled inflammatory markers and increased auto-fluorescence (AF) intensity at three different wavelengths, an effect blocked by inhibition of downstream signalling with Iκβ. *Ex vivo*, there was a significant increase in EPC number and AF intensity of individual EPCs from CVD patients concomitant with enhanced PECAM-1 expression when compared to normal controls. This novel label-free ‘lab on a disc’ (LoaD) platform can successfully detect endothelial activation in response to inflammatory stimuli *in vitro* and *ex vivo*.

## Introduction

The vascular endothelium lines the lumenal surface of blood vessels to provide an important non-adhesive and non-thrombogenic conduit for the cellular and macromolecular constituents of the blood under normal conditions^1^. Endothelial activation leading to dysfunction is a systemic pathological state of the endothelium that can be defined as an imbalance between pro-and anti-atherogenic substances produced by (or acting on) the endothelium and underlying smooth muscle^2^. Endothelial cell activation is defined by endothelial expression of cell-surface adhesion molecules, such as PECAM-1, VCAM-1, ICAM-1 and endothelial leukocyte adhesion molecule (ELAM, also known as E-selectin)^3^. Endothelial inflammation is a pivotal event in the pathogenesis of endothelial activation and is prevalent in many human diseases including atherosclerosis, septic shock, autoimmune diseases and ischemia/reperfusion damage^1^.

Among the numerous inflammatory mediators that contribute to endothelial activation leading to dysfunction, tumour necrosis factor alpha (TNF-α) is central to the initiation and termination of long-term inflammatory responses^4^. TNF-α binds to two type I transmembrane receptors, TNFR1 and TNFR2 that lack intrinsic catalytic activity and therefore require assembly of supramolecular complexes of cytosolic proteins to transduce their signals^5^. Briefly, activation of the inhibitor of kappa-B (IκB) kinases (IKKs) and their association with TNFR signalling leads to phosphorylation of the IκBα, and its ubiquitination and degradation^6^. In the absence of IκB, the p65- and p50-subunits of the nuclear factor (NF)-κB transcription factor (NFκB) enter the nucleus and bind to DNA to regulate transcription of key genes associated with the inflammatory response^6^. As a result, inflammation increases the interactions between endothelial cells and blood borne cells following the production of key adhesion molecules (ICAM-1, PECAM-1 and E-selectin) and their expression in endothelial cells^7–9^. In a similar manner, lipopolysaccharide (LPS) treatment induces endothelial cell activation and increased expression of adhesion molecules^10–12^.

Cellular oxidative stress, mediated by oxidised low-density lipoprotein (oxLDL), plays a pivotal role in endothelial cell inflammation and activation and the pathogenesis of atherosclerosis^13,14^. Oxidative stress and inflammation modulate circulating endothelial progenitor cells (EPCs) reparative functions on impaired/dysfunctional endothelium^15^ and the interplay between oxLDL induced inflammation and EPC biology as prognostic biomarkers of cardiovascular disease has attracted much recent attention^16^. Endothelial progenitor cells (EPCs) have also been implicated in liver injury and repair where increases in CD45^+^ EPCs, inflammatory cytokines and angiogenic mediators suggests an inflammatory role for these cells^17^.

Photonics has emerged as a powerful technology for contactless real-time analysis of biological samples in the life sciences and medicine^18^. Light as an interrogation mechanism has several major advantages including high sensitivity, non-destructive measurement, small or even non-invasive analysis and low limits of detection^19^. It is therefore an ideal technology platform to provide the next generation of diagnostic and prognostic tools. In combination with microfluidics, photonics facilitates and enables real time measurement of relevant analytes in very small sample volumes^20^. The light scattering properties (forward and side-angle scatter) of individual cells can be used to distinguish between discrete cell populations^19,21^. Forward light scatter has widely been used as an indicator of cell size and cell volume whereas side scattered light is influenced by nuclear morphology and cytoplasmic granulation reflecting the complexity of the internal structure of cells. The combination of forward and side scatter has been successfully used to identify specific cell types and subpopulations using flow cytometry^22^. In this context, altered light scattering properties associated with disease-induced cellular and molecular events may provide discrete photonic signatures that can be detected using label-free spectroscopic platforms *in vivo*^23,24^. Importantly, label-free prediction of DNA content and quantification of the mitotic cell cycle phases has recently been reported by applying supervised machine learning to morphological features extracted from brightfield and the typically ignored darkfield images of cells from an imaging flow cytometer^25^. This method facilitates non-destructive monitoring of cells avoiding potentially confounding effects of fluorescent stains while maximizing available fluorescence channels. In addition, the refractive index (RI) of cellular material also provides fundamental biophysical information about the composition and organizational structure of cells^26^, and in combination with photonic analysis has broad generic application in the study of cell growth and functional responses^27^.

**Figure 1:**
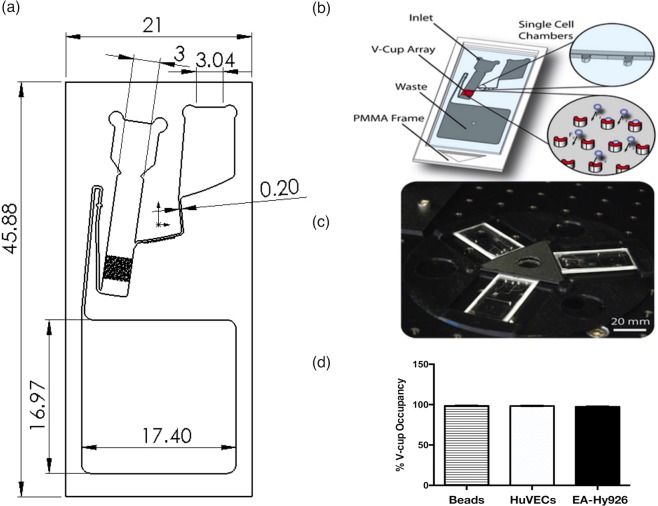
Label-free single cell optical profiling viewgraph. (**a**) Schematic of dimensions of the biochip device and channels. (**b**) Biochip schematic of operation [28]. (**c**) Three biochips under centrifugal microfluidic testing [28]. (**d**) V-cup array occupancy distribution for 20 µm polystyrene beads, HUVECs and EA.hy926 cells. Each test case shows an occupancy rate (one bead/cell per cup) of ≥95%. An empty V-cup or V-cup containing more than one bead/cell is classified as an error. (N = 10, p < 0.05, n = 1,128 V-cups). (**b**,**c**) Lab on a chip by Royal Society of Chemistry (Great Britain) Reproduced with permission of ROYAL SOCIETY OF CHEMISTRY in the format Journal/magazine via Copyright Clearance Center.)

We have previously developed and reported on a novel biochip design based on cell sedimentation under stagnant flow conditions due to the application of centrifugal force into an array of V-shaped capturing elements^28,29^ [Fig. 1a]. Briefly, the sedimentation takes place with the liquid bulk at rest to provide high capture efficiency. V-cups (13 μm diameter) staggered in an array of 47 × 24 cups can trap up to 1128 individual cells [Fig. 1b]. Additional single cell chambers (trap and pillar-based locations) are also on the biochip to facilitate a sub-population of cells to be selected (via optical tweezers) and further single cell assays. Finally, a disc for holding three biochips and for mounting onto the centrifugal test stand was manufactured using 3D printing [Fig. 1c] before the V-cup array occupancy distribution for 20 µm polystyrene beads, mammalian cells (HUVECs and EA.hy926 cells) was confirmed [Fig. 1d].

In the present study, we investigated whether a single cell label-free optical multi-parameter interrogation system could detect endothelial cell activation *in vitro* and *ex vivo*. FACS analysis was used to confirm endothelial cell activation in response to inflammatory stimuli *in vitro* while EPC number and PECAM-1 expression confirmed EPC activation *ex vivo*. Cells were then captured and interrogated using a label-free lab-on-a-disc ‘LoaD’ auto-fluorescence system that combines integrated optics with single-cell microfluidics^28^ to interrogate individual cells for differences across three different wavelengths.

## Results

### Validation of Endothelial Cell Activation in HUVECs and EA.hy296 cell populations

**Figure 2:**
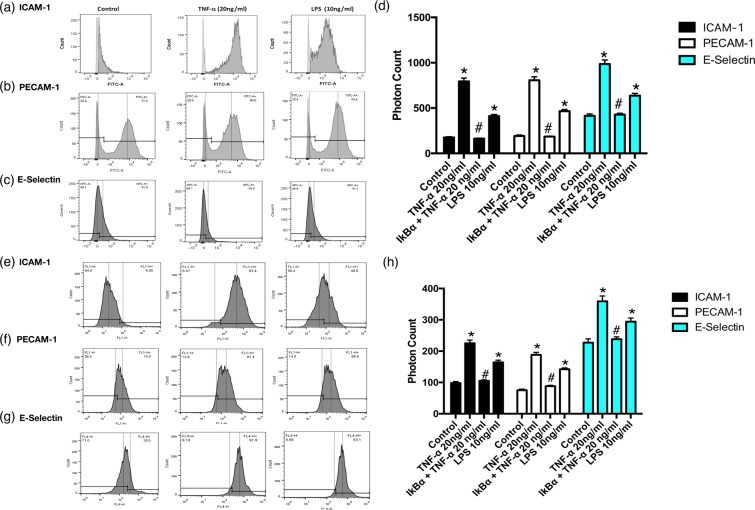
Flow Cytometric analysis of endothelial cell activation following TNF-α and LPS treatment. FACS analysis and photon counts of ICAM-1 (CD54), PECAM-1(CD31) and E-selectin (CD62) expression in (**a**–**d**) HUVECs and (**e**–**h**) EA.hy926 cells following treatment of cells with or without TNF-α (20 ng/ml) or LPS (10 ng/ml) for 24 hours respectively. (**d**) Inhibition of TNF-α induced endothelial activation and fluorescent photonic activity of PECAM-1, ICAM-1 and E-Selectin in HUVECs and (**h**) in EA.hy926 cells following NFκB inhibition with IκBα. (N = 3, p < 0.05, n = 10,000 cells).

Human umbilical vein endothelial cells (HUVECs) and an endothelial hybrid somatic cell line (EA.hy926) were both used in this study as established endothelial cell models^30^. In order to demonstrate that TNF-α and LPS both promote endothelial activation *in vitro*, HUVECs and EA.hy926 cells were exposed to TNF-α (20 ng/ml) or LPS (10 ng/mL) for 24 h before cells were fixed and analysed for inflammatory marker expression by FACS analysis. There was a marked increase in the proportion of HUVECs expressing ICAM-1 and PECAM-1 in cells treated with TNF-α and LPS [Fig. 2a–c] concomitant with a significant increase in the photonic fluorescent counts for PECAM-1, ICAM-1 and E-Selectin [Fig. 2d]. A similar profile was evident following treatment of EA.hy296 cells where the proportion of cells expressing the inflammatory markers ICAM-1 and PECAM-1 was increased [Fig. 2e–g] concomitant with an increase in the fluorescent intensities for PECAM-1, ICAM-1 and E-Selectin respectively [Fig. 2h]. Furthermore, inhibition of NF κB with IκBα treatment significantly attenuated the TNF-α-induced increase in fluorescent intensities for PECAM-1, ICAM-1 and E-Selectin in both HUVECs and EA.hy296 [Fig. 2d,h].

### Validation of Endothelial Activation in HUVEC and EA.hy296 single cell populations

**Figure 3:**
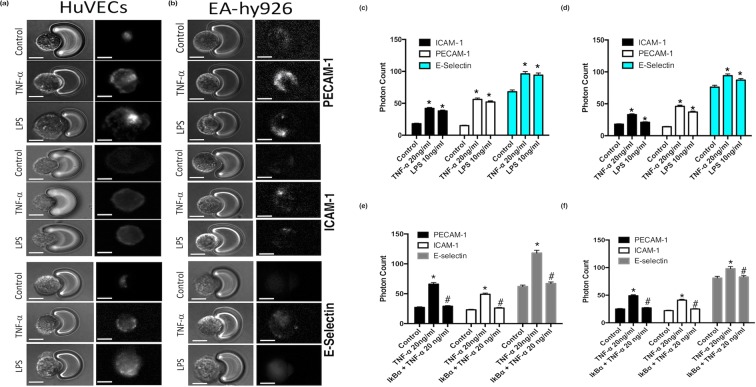
Immunocytochemical analysis of individual cells captured on the LoaD platform. (**a**,**b**) Representative immunocytochemical analysis of PECAM-1, ICAM-1 and E-selectin expression in individual HUVECs and EA.hy296 cells following treatment of cells with or without TNF-α (20 ng/ml) or LPS (10 ng/ml) for 24 hours. Bright field images and corresponding fluorescent images taken at 20x magnification with uniform region of interest selection for each image. (**c**,**d**) Cumulative analysis of the photonic fluorescence counts for PECAM-1, E-selectin and ICAM-1 following treatment with TNF-α and LPS, respectively. (**e**,**f**) Inhibition of TNF-α induced endothelial activation of PECAM-1 ICAM-1 and E-selectin in individual HUVECs and EA.hy296 cells, respectively, following NFκB inhibition with IκBα. (N = 3, *p = 0.035 ^#^p = 0.04 HUVECs, *p = 0.045 ^#^p = 0.04 EA.hy926 cells, n = 120 cells).

FACS analysis facilitates the acquisition of quantitative expression profiles for individual inflammatory markers across cell populations. We therefore assessed the inflammatory marker profile of individual single cells captured using the V-cup Biochip device using immunocytochemistry. Immunocytochemical staining for inflammatory markers PECAM-1, ICAM-1 and E-Selectin and the associated photonic profiles of individual cells were acquired and analysed. Representative immunocytochemical analysis of PECAM-1, ICAM-1 and E-Selectin expression in HUVECs and EA.hy296 revealed that both cell types exhibited enhanced immunofluorescent staining [Fig. 3a,b] while cumulative analysis of the photonic fluorescence counts for PECAM-1, E-selectin and ICAM-1 demonstrated that these markers were enhanced in both cell types following treatment with TNF-α and LPS, respectively [Fig. 3c,d]. Furthermore, inhibition of NFκB with IκBα treatment significantly attenuated the TNF-α-induced increase in photonic fluorescence counts for PECAM-1, ICAM-1 and E-selectin expression in both HUVECs and EA.hy296 [Fig. 3e,f].

### Single Cell Optical Auto-Fluorescence Profiles of Activated Endothelial Cells

**Figure 4:**
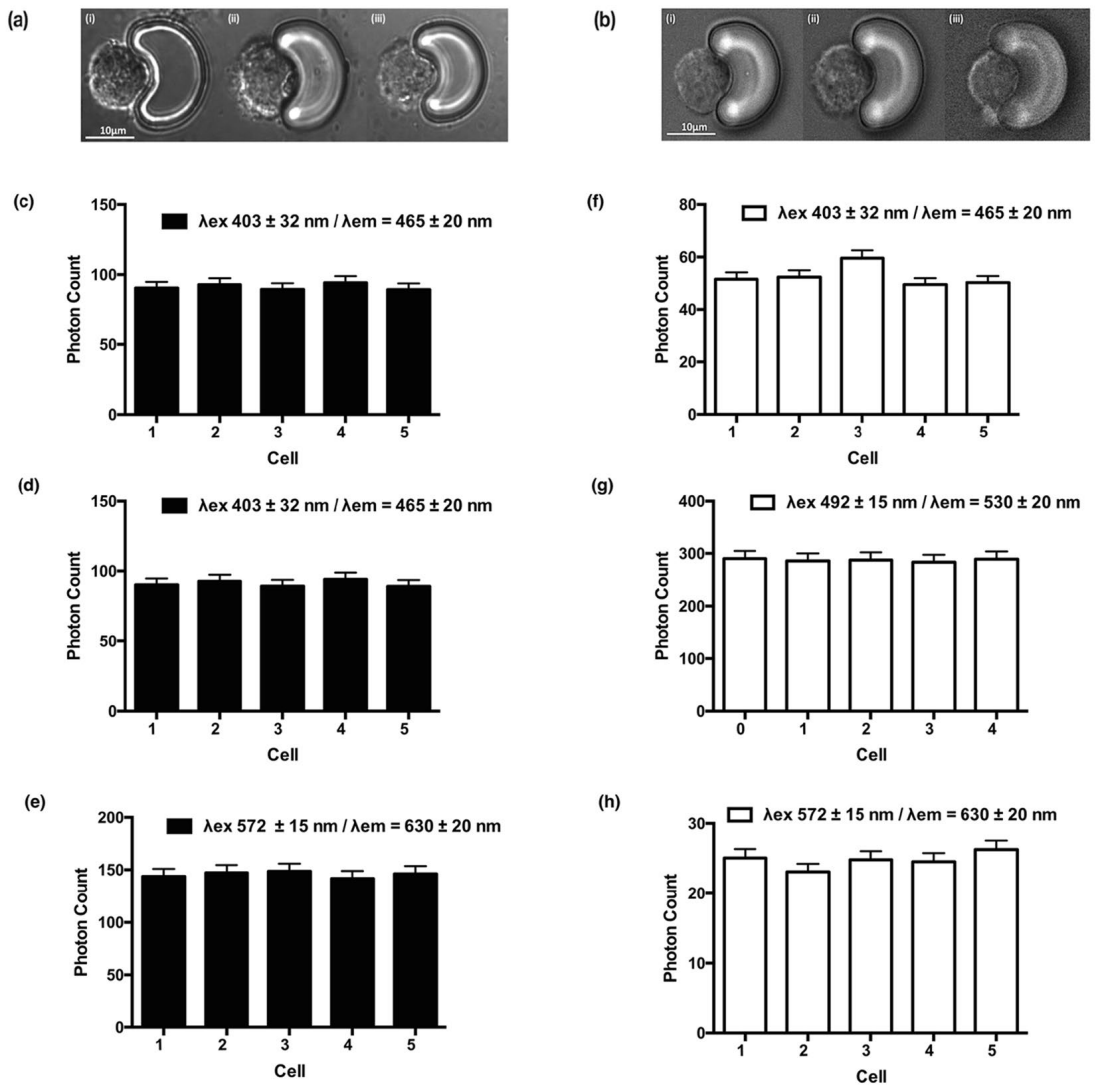
Auto-fluorescence (AF) analysis of individual cells captured on the LoaD platform. Stable Auto-fluorescence (AF) signal intensity across three wavelengths (465, 530 and 630 nm) of five individual cells (**a**) HUVECs and (**b**) EA.hy296 cells captured using the centrifugal ‘Load’ platform. (N = 3, *p = 0.035 HUVECs, *p = 0.04 EA.hy926 cells, bright field image magnification 20x.)

Initial single cell analysis (SCA) measurements on healthy HUVECs and EA.hy296 were acquired from cells individually arrayed onto scale-matched traps using the centrifugal Lab-on-a-Disc (LoaD) platform. Excitation was performed to allow both broadband light (λ = 360–800 nm) and selected fluorescent excitation (filtered at excitation wavelengths of 403 ± 32 nm, 492 ± 15 nm and 572 ± 15 nm) and emission wavelengths (emission filters currently used are 465 ± 20 nm, 530 ± 20 nm and 630 ± 30 nm), respectively. Bright field imaging was used to acquire data on the cell morphology and nuclei of individual cells [Fig. 4a,b]. Auto-fluorescence (AF) signals from five individual HUVEC and EA.hy296 cells were acquired. The AF spectra, provoked by the excitation wavelengths, consisted of three component bands with centre wavelengths at 465 nm, 530 nm and 630 nm. In HUVECS, the 530 nm showed the highest contribution in terms of AF intensity across the three wavelength bands examined, while in contrast, for EA.hy296 cells, the 465 nm band contributed the greatest AF intensities [Fig. 4e,g]. The analysis also confirmed that the AF intensities were highly consistent and generated discrete optical single cell signatures for each cell type in the relevant wavelength bands [Fig. 4c–e,f–h].

**Figure 5:**
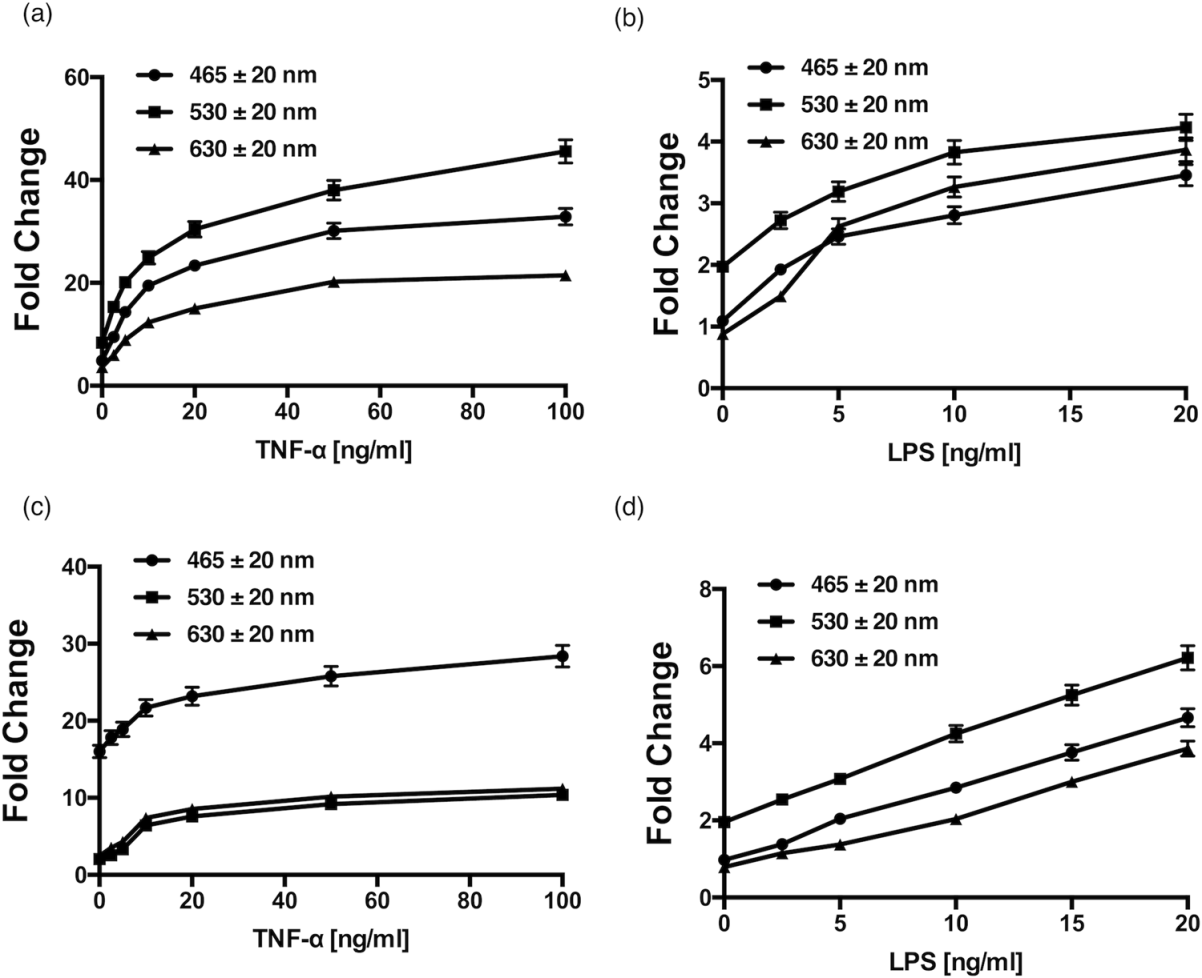
AF photonic analysis of endothelial cell activation following TNF-α and LPS treatment. The effect of increasing concentration of (**a**,**b**) TNF-α and (**c**,**d**) LPS on the auto-fluorescence (AF) signal intensity across three wavelengths (465, 530 and 630 nm) of individual HUVECs and EA.hy296 cells, respectively, captured using the centrifugal ‘Load’ platform. (N = 3, p < 0.05, n = 210 cells).

Treatment of HUVECs and EA.hy296 cells with increasing concentrations of TNF-α for 24 h produced a dose-dependent fold enhancement of the AF signature across three relevant wavelength bands [Fig. 5a,b], an effect mimicked by increasing concentration of LPS over the same time period [Fig. 5c,d]. Both TNF-α and LPS produced the most dramatic change to the 530 nm waveband in HUVECs [Fig. 5a,c]. Similarly, LPS produced the most dramatic increase in the 530 nm waveband in EA.hy296 cells [Fig. 5d]. In contrast, TNF-α produced the greatest change in the 465 nm waveband in EA.hy296 cells [Fig. 5b].

**Figure 6:**
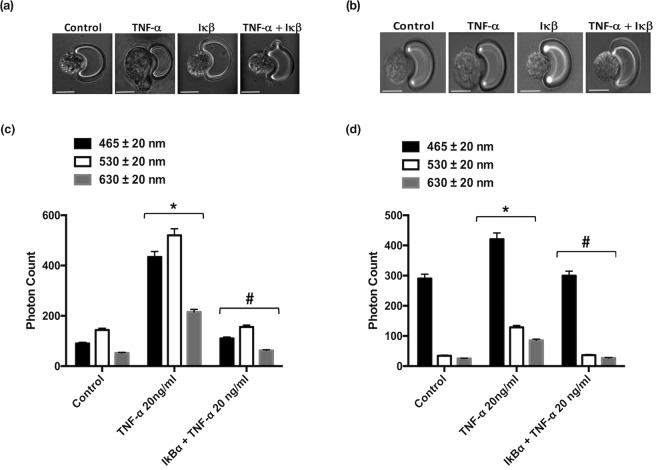
Morphological analysis of individual cells captured on the LoaD platform. Morphological changes of individual (**a**) HUVECs and (**b**) EA.hy296 cells following treatment with TNF-α in the absence or presence of IκBα for 24 h (N = 3, *p = 0.04, ^#^p = 0.045, n = 210 cells). Cumulative auto-fluorescence (AF) signal intensity across three wavelengths (465, 530 and 630 nm) of individual (**c**) HUVECs and (**d**) EA.hy296 cells following treatment with TNF-α in the absence or presence of IκBα for 24 h (N = 3, *p = 0.04, ^#^p = 0.045, n = 210 cells).

Treatment of HUVECs and EA.hy296 cells with TNF-α (20 ng ml^−1^) and LPS (10 ng ml^−1^) also produced a significant change in cell morphology concomitant with increased intensity over the three wavelength bands [Fig. 6 Pre-treatment of cells with IκBα prior to exposure of HUVECs or EA.hy296 cells to TNF-α resulted in a attenuation in the cell shape changes [Fig. 6 and Supplemental Fig. 1] concomitant with a reduction in the intensity of all AF signatures across the same three wavelength bands for both cell types, an effect also significantly attenuated following inhibition of NFκB with IκBα [Fig. 6c,d].Significant cell volume, shape and size (diameter) changes were observed as a result of treatment with TNF-α (20 ng ml^−1^) and LPS (10 ng ml^−1^) on both cell types (Supplemental Fig. 1).

**Figure 7:**
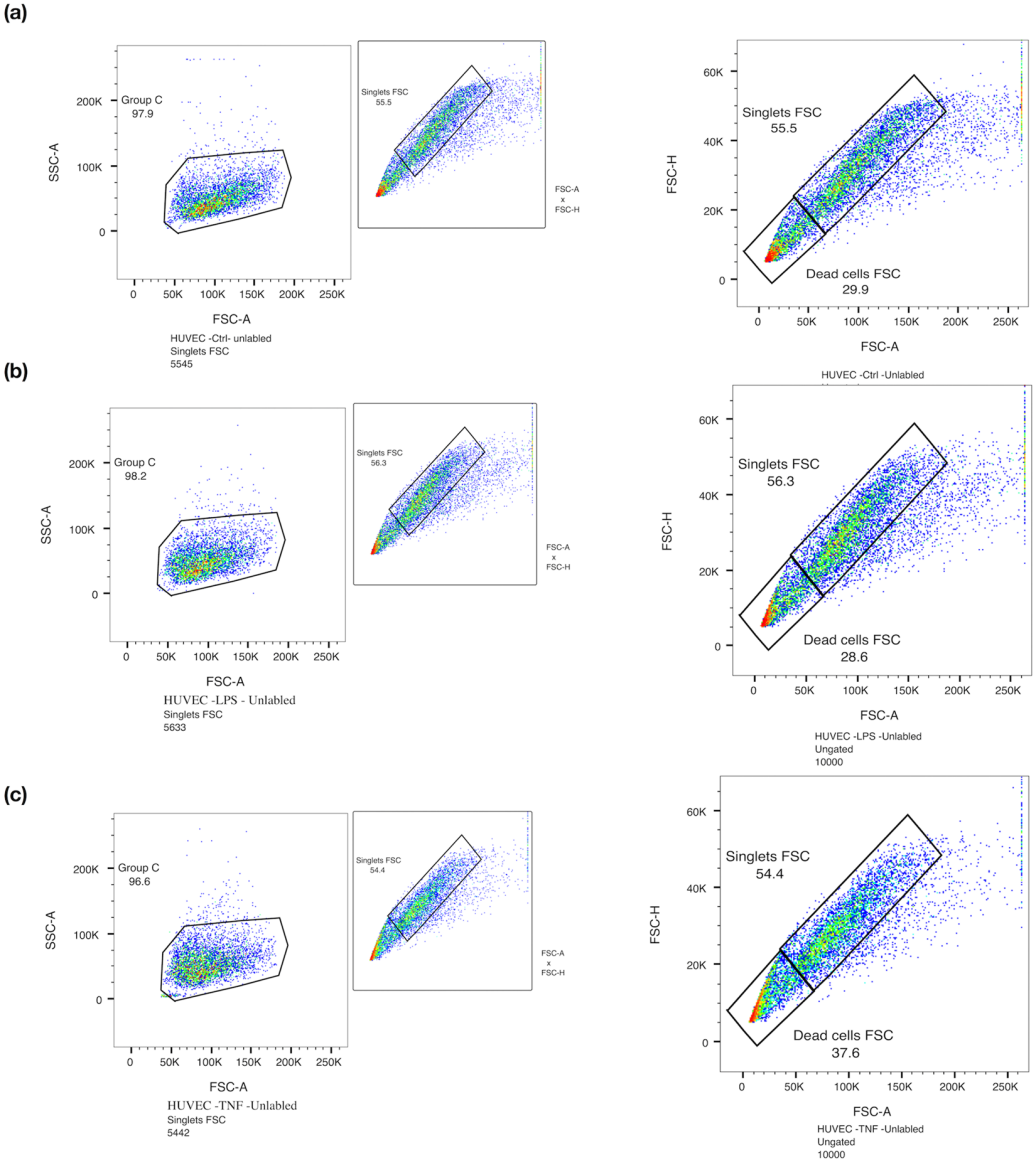
Label Free FACS analysis of endothelial cell activation following TNF-α and LPS treatment. Label-free FACS analysis of HUVECs following treatment with TNF-α and LPS, respectively. Cell populations as singlets were gated based on their internal complexity (SSC-H vs SSC-A) and based on their size (FSC-H vs FSC-A). (**a**) Control untreated cells, (**b**) TNF-α (20 ng/ml) and (**c**) LPS (10 ng/ml) for 24 hours. (N = 3, n = 10,000 cells).

In parallel studies, label free FACS analysis was also used to investigate the effect of TNF-α and LPS on activation endothelial cells. Cell populations as singlets were gated based on their internal complexity (SSC-H vs SSC-A) and based on their size (FSC-H vs FSC-A) [Fig. 7a–c]. Forward Scatter (FSC) is the amount of light scattered in the forward direction and is proportional to the cell size whereas side Scatter (SSC) is proportional to the internal complexity of a cell. Label free cells (control and activated) were then investigated further for cellular changes based on their size and cellular content (FSC-A vs SSC-A) within that gated singlet population. In contrast to single cell analysis (SCA), there was no significant determinable difference between the population of cells by label-free FACS analysis [Fig. 7a–c].

### Single Cell Refractive Index (RI)

**Figure 8:**
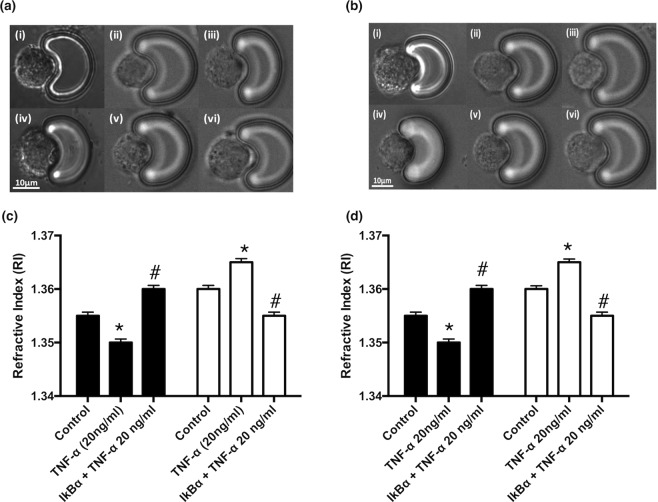
Endothelial activation and changes to the refractive index (RI) values of individual cells captured on the LoaD platform. The refractive index (RI) values for (**a**,**b**) cell membrane and (c, d) nucleus of (**a**,**c**) HUVECs and (**b**,**d**) EA.hy296 cells before and after TNF-α (20 ng/ml for 24 h) treatment in the absence or presence of IκBα for 24 h under similar index matching conditions (N = 3, p < 0.05, n = 210 cells).

Cell refractive index (RI) is a fundamental biophysical parameter that correlates with the intracellular contents, size and mass of a cell. Utilising opto-fluidic refractive index matching techniques with sucrose^26^, the corresponding RI values for the cell membrane and nucleus of HUVECs [Fig. 8a,c] and EA.hy296 cells [Fig. 8b,d] were determined before and after TNF-α treatment with or without inhibition of NFκB with IκBα treatment. Both cell populations underwent a reduction in the RI for the nucleus and an increase in the RI for membranes in response to the pro-inflammatory stimulus, under similar index matching conditions, an effect reversed following inhibition of NFκB with IκBα [Fig. 8c,d].

### Single Cell Optical Auto-Fluorescence Profiles of Activated Endothelial Progenitor Cells (EPCs)

**Figure 9:**
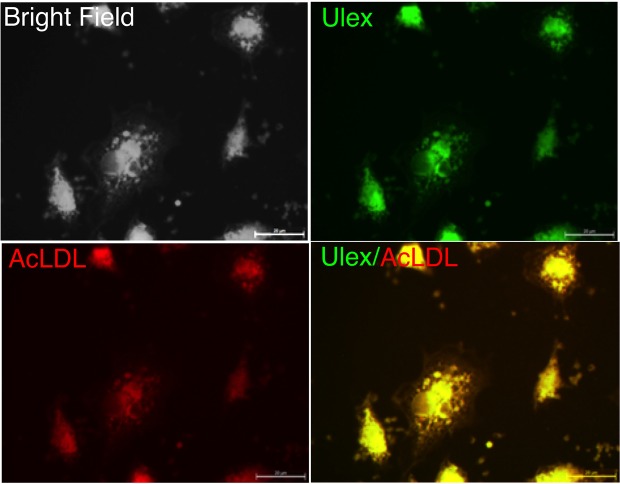
Double stained adherent endothelial progenitor cells (EPCs). Fluorescence microscopy (100x objective, bar corresponds to 20 µm) illustrates double-labelled adherent cells viewed in bright field (**a**). The cells were positive for binding of FITC-Ulex-lectin (**b**), and for uptake of Dil-labeled acetylated LDL (**c**). The staining in (**d**) show that the same cells stained for both FITC-Ulex-lectin and acetylated LDL and therefore agree with the current definition of cultured endothelial progenitor cells.

**Figure 10:**
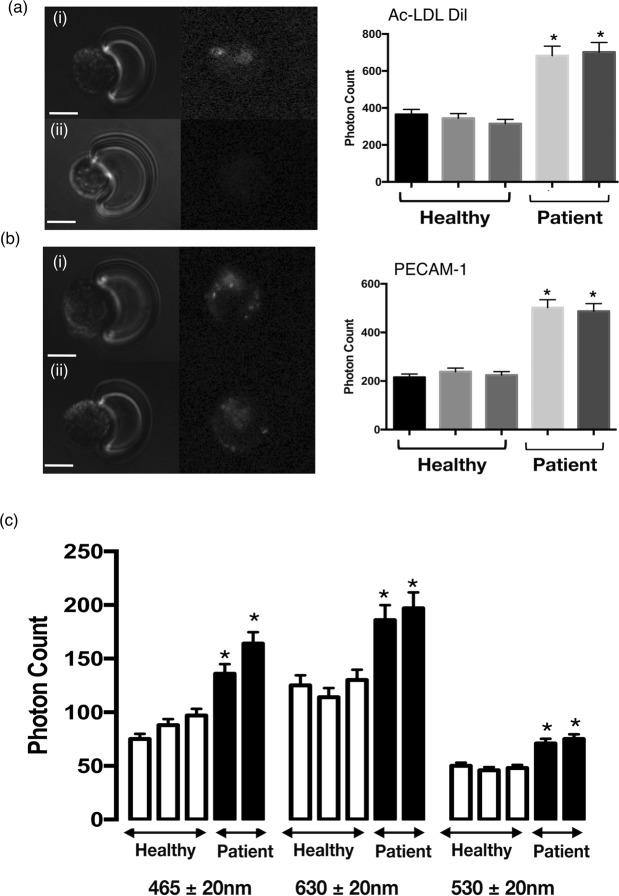
AF photonic analysis of endothelial progenitor stem (EPC) cell activation in normal and CVD patients. (**a**) Representative images ((i) CVD Patients and (ii) Healthy volunteers) and cumulative analysis of photon counts for AcLDL-DiI uptake. (**b**) Representative images ((i) CVD Patients and (ii) Healthy volunteers) and cumulative analysis of photon counts for PECAM-1 (Texas Red label) uptake. (**c**) AF signal intensity across three wavelengths (465, 530 and 630n) of individual captured EPCs from normal volunteers (Healthy) and CVD (Patient) patients, respectively. (N = 3, p < 0.05, n = 210 cells).

Total EPC number within mononuclear cells (MNC) isolated from peripheral venous blood was quantified as the number of cells that stain positive for Ulex lectin (UEA-1) and 1,1′-dioctadecyl-3,3,3′,3′-tetramethylindocarbocyanine perchlorate–acetylated low-density lipoprotein (AcLDL-DiI) [Fig. 9]. The photon intensity of AcLDL-DiI of individual captured cells and hence the number of EPC was significantly increased in patient samples when compared to healthy volunteer controls [Fig 10a,b]. Parallel analysis of immunocytochemical staining for the inflammatory marker, PECAM-1, and the associated photonic profile of individual cells confirmed that EPCs from patients exhibited a significantly higher photonic intensity for PECAM-1, when compared to normal healthy controls [Fig. 10b]. The AF spectra from individual EPCs consisted of three component bands with centre wavelengths at 465 nm, 530 nm and 630 nm, respectively. There was a significant increase in the AF intensity at each wavelength for the CVD patients, when compared to normal healthy volunteer controls [Fig. 10c].

## Discussion

The main finding of the present study is that TNF-α induced endothelial cell activation can be successfully detected label-free using a novel “Load” platform by measuring single cell autofluorescence (AF) and cell morphology *in vitro*. This novel label-free system was validated using both FACS analysis of cell populations and photonic analysis of individual ‘captured’ cells interrogated with specific fluorescent antibodies targeted against inflammatory markers. A comparative analysis of the LoaD technique versus established methods is summarised in Supplemental Table 1. These data clearly demonstrate that the AF profile of individual endothelial cells is a reliable platform to detect endothelial cell activation and may thereby facilitate the further development of label-free endothelial functional assays for use *ex vivo*. In this context, the AF intensity of individual EPCs from peripheral blood of CVD patients were significantly enhanced at 530 nm wavelength concomitant with enhanced PECAM-1 expression, when compared to EPCs from normal healthy volunteers.

Extensive experimental and clinical evidence supports a link between endothelial activation and inflammation^3^. Indeed, inflammatory cytokines are known contributors to the formation of atherosclerotic plaque and regulate key atherogenic responses throughout the atherosclerotic vessel^1^. The inflammatory response leads to endothelial activation as cells undergo cytoskeletal changes after exposure to an inflammatory stimulus that can last for several days. TNF-α secreted by lymphocytes and macrophages is one of the main cytokines involved in endothelial activation as they respond by increasing the levels of selectins and intercellular adhesion molecule-1 (ICAM-1) to promote the adhesion of monocytes^5,31,32^. Bacterial infections, associated with endothelial activation due to the innate immune response, increase the expression of pro-inflammatory cytokines like TNF-α and chemokines that also impact on endothelial function^10,33^. Lipopolysaccharide (LPS) stimulation of endothelial cells induces the expression of intercellular adhesion molecule-1 (ICAM-1), a critical molecule involved in the adhesive interaction between leukocytes and endothelial cells during the inflammatory response^12^. In this context, we used FACS analysis and immunocytochemical expression of key adhesion molecules in single cell HUVECs and endothelial hybrid somatic cells to model endothelial activation and activation *in vitro* and benchmark the label-free AF assessment of endothelial activation in single cells following exposure to TNF-α and LPS.

We confirmed that TNF-α and LPS both promote endothelial activation by increasing the expression levels of PECAM-1, ICAM-1 and E-selectin in both cell populations and in individual single cells. Moreover, the TNF-α inflammatory response was robust and was further attenuated following NFκB inhibition with IκBα. We provide compelling experimental evidence that inflammation and endothelial activation, which characterises endothelial dysfunction, can be classified based on the label-free AF photonic cell signatures acquired on a cell-by-cells basis. This single cell AF profile comprises of a set of label-free photonic signatures from cells individually captured and arrayed on scale-matched V-cup traps using a centrifugal Lab-on-a-Disc (LoaD) platform. Inflammation in endothelial cells induced by two dissimilar pro-inflammatory stimulators, TNF-α and LPS, can be detected and this signature was characterised by sharp increases in three broadband wavelengths, 465 nm, 530 nm and 630 nm, respectively. Moreover, the TNF-α induced AF signature was further attenuated following NFκB inhibition with IκBα confirming that the signature is specific to NFκB-induced events within an individual endothelial cell.

The changes in AF intensity in response to both pro-inflammatory stimulators were dose-dependent but dissimilar in the extent of the changes. This might reflect the subtle differences in how these two pro-inflammatory stimuli promote inflammation and endothelial activation. Indeed, exposure to TNF-*α* following activation of its transmembrane receptors, TNFR1 and TNFR2, triggers several signalling cascades in HUVECs, especially NFκB, c-Jun N-terminal kinase (JNK), and p38 mitogen-activated protein kinase pathways, leading to the production of pro-inflammatory cytokines^7^. In addition, TNF-α can reorganize the F-actin cytoskeleton of endothelial cells, leading to the formation of stress fibres^34^ and modulate cell permeability by enlarging intercellular gaps, promoting vascular leakage at sites of inflammation^35^. In contrast, while LPS induces many similar intracellular responses, including activation of nuclear factor-κB (NF-κB) and activation of members of the mitogen-activated protein kinase (MAPK) family^33^, it also binds the receptor of advanced glycation end products (RAGE), a member of the immunoglobulin super family to promote inflammation^36^. The activation pattern between TNF-α and LPS exhibits qualitative differences, primarily the special localization of Toll-like 4 (TLR4)^11^ and differences in the kinetics of the signalling pathways of TNF-α and LPS. The most striking dissimilarity is the reported low expression levels of IL-6 in response to TNF-α compared with that of LPS. In addition, several groups have reported differences in the ability of LPS and TNF-α to induce transcriptionally regulated adhesion molecules and cytokines, in part due to significant dissimilarity in the promoter regions of ICAM-1, E-selectin and other pro-inflammatory adhesion molecules^37^. Collectively, these subtle differences may be responsible for divergence in the AF photonic signature of cells before and after exposure to these pro-inflammatory stimuli.

TNF-α and LPS are thought to promote endothelial activation and dysfunction by inducing oxidative stress^14,38^. Malondialdehyde (MDA) generated in the oxidative degradation process of polyunsaturated lipids is an active modifying agent of proteins both *in vitro* and *in vivo* and is regarded as a biomarker of oxidative stress^39^. As a product of lipid peroxidation, MDA accumulates during many pathophysiological processes, including inflammation^40^. Therefore, MDA and MDA-modified (adducted) proteins may be responsible for the observed enhanced AF signatures following endothelial activation. Malondialdehyde-acetaldehyde (MAA) adducted proteins are capable of inducing endothelial cells to produce and release TNF-α, and cause up-regulation of endothelial adhesion molecule expression, including ICAM-1^40^. Alternative molecules likely responsible for AF changes following endothelial activation include many cellular metabolites that exhibit autofluorescence^41^. Flavin, a ubiquitous organic compound involved in the metabolism of most organisms and capable of undergoing oxidation-reduction reactions is auto-fluorescent, as are derivatives of riboflavin^41,42^. Flavin oxidase-induced ROS generation is known to mediate dose-dependent endothelial cell damage^43^. Other common species include nicotinamide adenine dinucleotide (NADH) and its derivatives, which are crucial to endothelial cellular integrity and signalling^44^. Less-well-known sources may include lipofuscin, a substance found to positively stain for lipid, carbohydrate and protein that may cause oxidative and photooxidative damage through its phototoxic properties as it progressively accumulates in cells^45^. Although further studies will be required, taken together the evidence suggests that oxidative stress may lead to the changes underlying the increased autofluorescence observed in the present study. The changes in autofluorescence by themselves appear sufficient for rapidly measuring changes in endothelial cell state by label-free single cell analysis.

The refractive index (RI) reveals a unique aspect of cellular structure, and is important in studies of cell and tissue light scattering, laser trapping of single cells, flow cytometry, total internal reflection microscopy and other areas involving the interaction of light with cells and tissues^26^. Several different methods have been developed to measure the effective refractive index of a single cell. Immersion refractometry exploits the intensity contrast between a cell and its surrounding medium using phase contrast microscopy whereby the cell appears invisible when its effective refractive index matches with that of the surrounding medium^46^. By refractive index matching with sucrose, endothelial cells exposed to a pro-inflammatory stimulus increased their RI index values for membranes but decreased their value for nucleus under similar matching conditions. Similar changes in RI have been reported for macrophages following infection^47^. Furthermore, Digital holographic microscopy (DHM) assessment of RI demonstrated a strong correlation between the severity of inflammation and the RI within the colon^48^. These data suggest that a reduction in the RI of the nucleus due to the changes in the nucleus of endothelial cells may be useful for general real-time monitoring of endothelial activation and dysfunction by pro-inflammatory stimuli.

The main limitation of the present approach is that the spectral measurements were made on single cells, rather than being obtained in an intact endothelial cell layer *in situ*. However, endothelial progenitor cells and circulating endothelial cells can be retrieved from blood for clinical evaluation^49^ and a suitable technology is required to provide a definitive tool for diagnosis of endothelial function in patients. In this context, the AF intensity of individual EPCs from peripheral blood of a small sample of CVD patients were significantly enhanced at 530 nm wavelength concomitant with enhanced PECAM-1 expression, when compared to EPCs from normal healthy volunteers. The distinctive advantage of this technology is a highly efficient cell-to-light interaction, facilitating the direct detection of dysfunctional endothelial cells by measuring the optical broadband wavelength photonic profile of a cohort of individual cells. This possibility was validated using two sources of endothelial cells following pro-inflammatory induction of endothelial activation *in vitro* and individual EPCs from peripheral blood of a small sample of patients with hypertension and breast cancer *ex vivo*.

In conclusion, we present a novel label-free photonics approach using a LoaD platform to provide a highly reproducible enabling technology that has the potential for development as an early detection platform for interrogating endothelial activation by inflammatory stimuli *ex vivo*.

## Methods

### Cell Culture

Human umbilical vein endothelial cells (HUVECs) were obtained from PromoCell GmbH, Germany (C-12203 HUVEC-c pooled). Cells were cultured in PromoCell Endothelial Cell Growth Medium (C22110), supplemented with Fetal Calf Serum (0.02 ml ml^−1^), Endothelial Cell Growth Supplement (0.004 ml ml^−1^), Epidermal Growth Factor (recombinant human) (0.1 ng ml^−1^), Basic Fibroblast Growth Factor (recombinant human) (1 ng ml^−1^), Heparin (90 µg ml^−1^), and Hydrocortisone (1 µg ml^−1^). Cells were cultured in 75-cm^2^ flasks at 37 °C and 5% CO_2_. When harvesting or passaging the HUVEC cells, the PromoCell Detach Kit (C41200) was used. Cells were isolated by centrifugation at 220 x g for 3 minutes. The cell pellet was finally re-suspended in culture media. Cells analysed were cultured from four independent lot numbers to account for cell batch variations.

The human umbilical vein cell line, EA.hy926, was established by fusing primary human umbilical vein cells with a thioguanine-resistant clone of A549 by exposure to polyethylene glycol (PEG). EA.hy926 cells were obtained from ATCC USA (ATCC CRL-2922), and cultured in Dulbeccos Modified Eagles Medium (DMEM) supplemented with 10% Fetal Bovine Serum (FBS). Cells were cultured in 75-cm^2^ flasks at 37 °C and 5% CO_2_. For harvesting or passaging the cells, standard trypsinisation protocols were used. Briefly, culture media was removed and the cells were washed in 5 ml PBS. The PBS was removed and 4 ml of 0.25% Trypsin/0.1% EDTA was added for 5 minutes at 37 °C. 4 ml culture medium was added to neutralize the trypsin, and cells were isolated by centrifugation at 300 × g for 4 minutes. The cell pellet was re-suspended in culture media. Cells analysed were cultured from two independent lot numbers to account for cell batch variations.

### Isolation and assessment of EPC number

Mononuclear cells (MNC) were isolated from a sample 20 ml of citrated peripheral venous blood from representative normal healthy volunteers and one breast cancer and one hypertensive patient by density centrifugation using Histopaque-1077 according to the manufacturer’s instructions. Cells were seeded at a density 2 × 10^6^ cells/well and cultured in EBM-2 + EGM-2-MV-Single Quots medium containing 10% FBS. Total EPC number was quantified as the number of cells on a coverslip that stain positively with Ulex lectin (UEA-1) and 1,1′-dioctadecyl-3,3,3′,3′-tetramethylindocarbocyanine perchlorate–acetylated low-density lipoprotein (AcLDL-DiI) 5 days after plating. After staining, samples were viewed using a fluorescence microscope (Zeiss) and a representative photograph of a high-power field taken for documentation purposes. Dual-stained cells positive for both lectin and AcLDL-DiI were judged to be EPC. The number of EPC per well will be evaluated by counting and averaging the results from 3 randomly selected high-power fields.

### Endothelial Activation *in vitro*

Endothelial activation was induced by exposing the cells to various concentrations of TNF-α (50103 ab9642 Active TNF alpha full length protein, Abcam Ltd.) for 24 h. TNF-α was diluted to a final stock concentration of 200 μg ml^−1^ in dd.H_2_O with 0.1% BSA and filtered through a 0.22-μm filter. IκBα is an inhibitor of the NFκB complex and was used to prevent TNF-α induced inflammation^50^. IκBα (SRP5195-50UG, Sigma Aldrich Ireland) and was diluted in dd.H_2_O with 0.1% BSA and filtered through a 0.22-μm filter to a final stock concentration of 200 μg ml^−1^. A second method of endothelial activation was implemented by exposing the cells to various concentrations of LPS (lipopolysaccharide, L2654, Sigma Aldrich, Ireland) for 24 h. The LPS solution was diluted in dd.H_2_O with 0.1% BSA and filtered through a 0.22-μm filter to a final stock concentration of 1 mg ml^−1^.

### Biochip Device

The base (microfluidic inlets and V-cup array) section of the biochip was fabricated in PDMS (Sylgard 184, Dow Corning GmbH, Germany). Moulds for PDMS casting were surface micro-machined using SU8-3025 (Microchem, USA) for manufacturing the V-cup array and the reservoirs. The biochip middle layer (chip support holder) was manufactured using poly(methyl methacrylate) (PMMA) with a thin layer of pressure sensitive adhesive (PSA) attached to its base. A laser cutter (Epilog Zing Laser, Epilog, USA) defined the middle layer of the biochip. The chip substrate consisted of a standard borosilicate microscope slide which was bonded to the chip middle layer using PSA. This hybrid chip was then treated by air plasma (1000 mTorr) for 5 minutes and assembled with the PDMS base to complete the biochip^28^. The fabrication method for the biochip ensured transparency and biocompatibility and was leak free and facilitated long term stability. The operating principles of the V-cup array have been described previously^29^.

The centrifugal test setup comprised a motor for spinning the microfluidic chips (4490H024B, Faulhaber micromotor SA, Switzerland), a synchronized camera for image acquisition during rotation (TXG14c, Baumer AG, Germany) coupled to a motorized 12x zoom lens (Navitar, USA) and a strobe light unit (Drelloscop 3244, Drello, Germany) as described previously^28^. The system integration between the microfluidic and optical systems for SCA was performed using an in-built optical detection and imaging system on the centrifugal test stand. The optical module incorporated a laser tweezers to manipulate individual cells on disc using a 1-W, 1064-nm infrared laser (Roithner Lasertechnik, Austria). This laser was focused through a 40x oil immersion microscope objective (CZ Plan Neofluar 40 ×/1.3 OIL PH3, Zeiss, Germany) with a numerical aperture (NA) of 1.3. This setup allowed a working distance of 200 μm. This objective was mounted on a piezo driven Z-drive with a travel range of 100 µm (Fast PIFOC® Piezo Nanofocusing Z-Drive, PI, Germany) for fine focusing. Additionally, the module included a high sensitivity cooled CCD camera (Sensicam qe, PCO, Germany) which utilizes the same optical path as the laser to facilitate particle handling and acquisition of bright-field and fluorescent images. Excitation was performed by a 250-W halogen lamp (KL 2500 LCD, Schott, Germany) with an enclosed filter wheel to allow both broadband light (λ = 360–800 nm) and selected fluorescent excitation (filtered at excitation wavelengths of 403 ± 32 nm, 492 ± 15 nm and 572 ± 15 nm) and emission wavelengths (emission filters currently used are 465 ± 20 nm, 530 ± 20 nm and 630 ± 30 nm). The module was mounted on a computer controlled X-Y stage (Qioptiq, Germany).

### Biochip Preparation and Microfluidic Testing

The device was placed in vacuum prior to introducing the liquids for a minimum of 30 minutes ensure complete and bubble-free priming. The biochip was primed with PBS buffer with 1% BSA (bovine serum albumin) via the loading chamber on the top right section of the biochip. After priming, cells were introduced via the loading chamber on the top left section of the biochip. All pumping was performed the centrifugal test stand and a 3D-printed chip holder which allows three biochips to be tested in parallel, thus significantly increasing the cell capture efficiency of the V-cup array system compared to common, flow-driven systems^28^. Initial cell capture tests were performed using 20-μm polystyrene beads to emulate cell behaviour before being repeated using HUVECs, EA.hy926 cells and human EPCs. In all cases, the sedimentation in absence of flow led to significantly increased occupancy of the V-cups (≥95%) compared to common, flow-driven methods (Fig. 1). Cell capture is undertaken at a spin speed of 10 Hz for a duration of 30 minutes. Cells are then immediately imaged for a period ranging from 45–90 minutes upon completion of cell capture.

The maintenance of cell viability on chip has also been studied for both HUVECs and EAhy926 cells (Supplemental Fig. 2). Post capture, cells have been incubated with a Live/Dead cell imaging kit (R37601, Thermo Fisher Ireland) and viability measurements made at hourly intervals over twelve hours based on the uptake of cell-impermeable dye for staining of dead and dying cells, which are characterized by compromised cell membranes.

### Refractive Index (RI) Matching

The RI of the cell membrane and nucleus was determined by index matching by replacing the standard running buffer (PBS with 1% BSA) on chip around cells captured on the V-cup array with various sucrose concentrations^26^. By varying the density and percentage weight by volume of a sucrose solution, the RI of the solution can be tuned to a desired value^26^. The working principle of immersion refractometry is based on imaging by a phase contrast microscope. When the external sucrose based buffer medium has a refractive index higher than the cell nucleus or membrane, the cell region of interest appears darker. Whereas when the external sucrose buffer medium has a lower refractive index, the cell nucleus or membrane brighter. Once the refractive index of the buffer medium is equal to the one of the cell nucleus or membrane, the cellular region of interest is invisible. Hence, this null method can be employed to measure the refractive index of the cell nucleus or membrane under test.

### Immunocytofluorescence

Both HUVECs and EA.hy926 cells were isolated by centrifugation at 220 g for 3 minutes. The culture media surrounding the resulting cell pellet was removed and replaced by 1 ml of PBS with 1% BSA. Cells are then fixed using a 4% solution of formaldehyde (Thermo Scientific Product Number 28906). A 5-μl volume of Alexa Fluor® 594 anti-CD31 PECAM-1 (Biolegend Product Number 303126**)**, Alexa Fluor® 488 anti-CD54 ICAM-1 (NovusBio Product Number DDX0150A488) and Alexa Fluor® 488 anti-CD62E E-selectin (Biorbyt Product Number orb170889) was added and then incubated on a rotational plate for a duration of 1 hour. Upon completion of the incubation period, the cells were then twice isolated by centrifugation at 220 g for 1 minute and washed in fresh PBS with 1% BSA. Cells were then loaded on to the biochip and imaged with an Olympus IX81 motorized inverted fluorescence microscope with an attached Hamamatsu ORCA - ER digital camera C4742-80 using a 10x and 20x objective.

### Flow cytometry

The cells were prepared, fixed and incubated with FITC labelled anti-CD31 PECAM-1 (Biolegend Part Number 303104), Alexa Fluor® 488 anti-CD54 ICAM-1 (Biolegend Part Number 322714) and Alexa Fluor® 647 anti-CD62E E-Selectin (Biolegend Part Number 148801) according to the manufacturer’s instruction. Flow cytometry was performed using a BD FACS Calibur. All analyses were performed using FlowJo software (Tree Star Inc., 92 Ashland, OR, USA). Cytometry data was gated to remove cell debris and doublets. Cells defined to be expressing the antibody were defined as FITC positive and the percentage of FITC positive cells in each of the test subpopulations was determined.

### Data analysis

Images were analysed by the open-source software ImageJ (version 1.46r). Cell signal intensity measured in photon counts from the detection CCD camera was quantified by ‘region of interest’ (ROI) analysis. Background signal levels are removed and an average intensity was obtained for each cell through the time series. All results are reported as mean values ± standard errors (where s = standard deviation and n = number of cells from at least three independent experiments). Statistical differences were analysed by one-way analysis of variance (ANOVA) and probability levels below 5% were considered significant.

## Supplementary information

Supplementary Information
