# Supplementary material for: Label-Free Multi Parameter Optical Interrogation of Endothelial Activation in Single Cells using a Lab on a Disc Platform
Source: Sci Rep. 2019 Mar 11;9:4157. doi: 10.1038/s41598-019-40612-8 (PMC6411894; doi:10.1038/s41598-019-40612-8)
Supplement: Supplementary file 1 — Supplementary Information [file 41598_2019_40612_MOESM1_ESM.pdf]

Label-Free Multi Parameter Optical Interrogation of Endothelial Activation in Single Cells using a Lab on a Disc Platform

Damien King[1], MacDara Glynn[1], Sandra Cindric[1], David Kernan[1], Tríona O'Connell[2], Roya Hakimjavadi[3], Sinéad Kearney[1], Tobias Ackermann[3], Xavier Munoz Berbel[4], Andreu Llobera[4], Ulf Simonsen[5], Britt E Laursen[5], Eileen M Redmond[6], Paul A. Cahill[3] and Jens Ducrée*[1]

[1]Dublin City University, School of Physical Sciences, National Centre for Sensor Research (Dublin, Ireland);

[2]Dublin City University, School of Biotechnology, Irish Science Separation Cluster (Dublin, Ireland);

[3]Dublin City University, School of Biotechnology, Vascular Biology & Therapeutics (Dublin, Ireland)

[4]Centre Nacional de Microelectronica, Campus UAB (Barcelona, Spain);

[5]Aarhus University, Department of Biomedicine (Aarhus, Denmark);

[6]University of Rochester, Dept Surgery (Rochester, New York, United States);

***Please address correspondence to:**

Prof Jens Ducrée, Dr. rer. nat. habil. Dipl. Phys.
Fraunhofer Project Centre for Embedded Bioanalytical Systems
DCU - Dublin City University
School of Physical Sciences
Glasnevin
Dublin 9
Ireland
Phone: +353 1 700 7658 / 5299
Fax: +353 1 700 7873
email: jens.ducree@dcu.ie
http://www.dcu.ie/fpc
http://www.dcu.ie/microfluidics/

Supplementary Information

SI Table 1: Comparison of state-of-the-art technologies for the LoaD single cell analysis system

Technology	Key Features (USPs)	Strengths	Weaknesses	Other
LoaD Single Cell Analysis System	• Single cell resolution<br>• Real time measurement<br>• Sequential experiment capability	• Sedimentation based cell capture (minimal shear flows)<br>• Time stamped imaging<br>• Rapid fluorescence detection<br>• Single cell isolation	• Low – medium throughput<br>• Not validated in industrial settings	• Mixed cell population handling<br>• Low cell number handling capability<br>• Test volume 8µl
Flow Cytometry	• Established industry method<br>• Rapid method<br>• High throughput	• Quantitative<br>• Employed in industrial and clinical environments<br>• Industry gold standard method	• Large cell number required (n = 10,000)<br>• Population level analysis (sub-population versus single cell)<br>• Cells exposed to high shear pressures<br>• Cannot correlate retrieved cells to FACS data for further validation	• End point measurements<br>• Complex data analysis<br>• High cost systems (€300,000 +)<br>• Regular maintenance required<br>• Trained operators required<br>• Test volume 500µl
Highly Integrated Cellular Analysis Systems	• Validated for industrial and clinical applications<br>• Multiple parameter single cell analysis	• High throughput<br>• Confirmatory<br>• Real time imaging<br>• Single cell resolution<br>• Integrated analysis (Qp, IgG production, growth rate, viability, morphology)	• Very high system cost (>> €1,000,000)<br>• Labour intensive	• Highly Skilled operators required
Cell Viability Instrumentation	• Established industry method<br>• Rapid method<br>• Combinatorial FACS & Imaging approach	• High throughput<br>• High cell concentration handling<br>• Multiple parameter measurements (VCD, cell count etc.)	• High system cost<br>• Large test volume 1ml	• Viability range: 20% - 100%
Single Cell Printers / Dispensers	• Inkjet printer principle<br>• Rapid single cell seeding	• Automated single cell imaging and dispensing<br>• Compatible with multiple well plate formats (96, 384)	• Limited to cell dispensing and imaging<br>• No sequential measurements<br>• Doublet based droplets discarded<br>• Cells exposed to nozzle based pressure system	• Droplet based technology

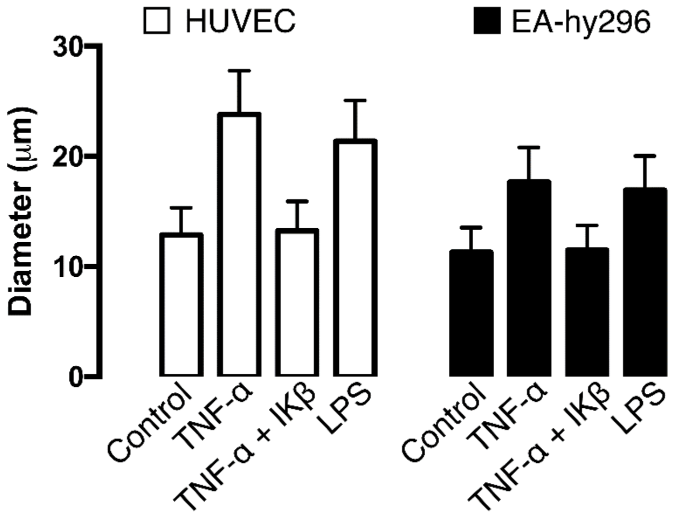

SI Figure 1: Cell size comparison of HUVECs and EA.hy926 cells post treatment. Cell shape and size (diameter) changes were observed as a result of treatment with TNF-α (20ng ml^{-1}) and LPS (10ng ml^{-1}) on both cell HUVEC and EA.hy926 cell types. (N=3, $p<0.05$, n= 25 cells).

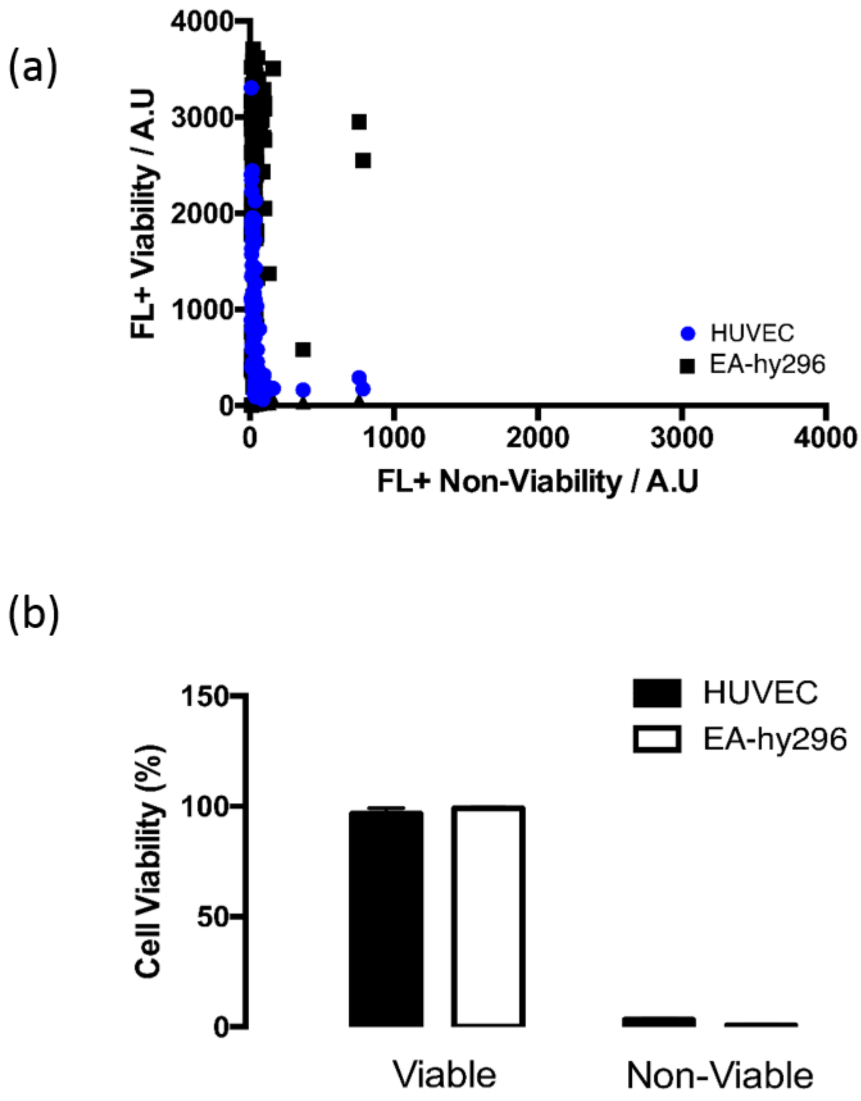

SI Figure 2: Maintenance of cell viability on chip (post capture and analysis). Cell viability measurements made at hourly intervals over twelve hours based on the uptake of cell-impermeable dye for staining of dead and dying cells, which are characterized by compromised cell membranes. (a) Single cell viability plots and (b) overall percentage cell viability on chip are presented. (N=3, $p<0.05$, n= 125 cells).
